# Underrepresentation of Black Men in Physician Assistant and Associate Training

**DOI:** 10.1001/jamanetworkopen.2024.41531

**Published:** 2024-10-28

**Authors:** Lucy W. Kibe, Katrina M. Schrode, Samuel Paik, Dominique Frias-Sarmiento

**Affiliations:** 1 Physician Assistant Program, Charles R. Drew University of Medicine and Science, Los Angeles, California; 2 Department of Psychiatry, Charles R. Drew University of Medicine and Science, Los Angeles, California; 3 PA (Physician Assistant/Associate) Education Association, Washington, DC

## Abstract

**Importance:**

The underrepresentation of Black men in the health care workforce, particularly among physician assistants and associates (PAs), represents a missed opportunity to alleviate shortages of health care professionals and enhance workforce diversity. Increasing the number of Black men in the training pipeline could significantly benefit education, patient care, and overall health outcomes.

**Objective:**

To analyze patterns from 2013 to 2021 among Black men applying to and matriculating into PA programs and compare these figures with the age-specific US population of Black men.

**Design, Setting, and Participants:**

This cohort study assessed deidentified application and matriculation data from the Central Application Service for Physician Assistants (CASPA) for PA training programs in the US, alongside US Census data. CASPA applicants reporting race and gender identities as Black male were included. Data were obtained March 10, 2023, from the 2012-2013 to the 2020-2021 application cycles; data were analyzed from June 2023 to May 2024.

**Exposures:**

Black male applicants and matriculation.

**Main Outcomes and Measures:**

Patterns and proportion of Black male applicants and matriculants to PA programs relative to total rates and expected numbers.

**Results:**

From 2013 to 2021, PA programs grew 64.3%, with an increase in applicants from 19 761 to 30 196 and matriculants from 6192 to 11 115. Despite this growth, the representation of Black men among applicants (from 435 to 732) and matriculants (from 73 to 156) remained stagnant, averaging 2.2% and 1.2%, respectively, significantly lower than their 8.7% (3 920 231 individuals aged 20-29 years) representation in the US Census. In 2021, the matriculation rate for Black men (156 of 732 [21.3%]) was notably lower than the matriculation rate for all applicants (11 115 of 30 196 [36.8%]). Based on the expected proportion of Black men in the PA applicant age range, there should have been 2641 applicants (actual: 732) and 972 matriculants (actual: 156) across 308 PA programs in 2021; these numbers indicate that, per program, approximately 9 applicants would have to have been evaluated and 3 matriculated to achieve parity, in contrast to the mean of 2 applicants and 1 matriculant per 2 programs.

**Conclusions and Relevance:**

In this cohort study of PA applicants and matriculants, Black men remained substantially underrepresented despite overall growth of PA training programs. The low representation among matriculants was due in part to the low numbers of applicants but also to substantially lower matriculation success. This persistent underrepresentation highlights systemic barriers and underscores the need for targeted interventions to achieve a more representative health care workforce. To achieve equitable admissions, each PA program should aim to evaluate 9 Black male applicants and matriculate 3 Black men annually.

## Key Points

**Question:**

Have Black men had equitable access to physician assistant and associate (PA) training programs in the past decade?

**Findings:**

In this cohort study, despite the profession’s significant growth, Black men aged 20 to 29 years represented only 2.2% of applicants and 1.2% of matriculants in the PA profession from 2013 to 2021, substantially lower than their 8.7% representation in the age-specific US population.

**Meaning:**

These findings suggest that the PA profession has made no progress in increasing the numbers of Black men in PA training programs and that to achieve population parity, each PA training program should evaluate more applicants and matriculate more Black men annually.

## Teaser

This cohort study examines the pipelines that funnel Black men to the physician assistant or associate workforce and disparities in applications and matriculation compared with other groups in US programs.

## Introduction

The US health care system has witnessed substantial growth and transformation in the last few decades, with an ever-increasing demand for skilled health care professionals. As the Baby Boomer generation ages, the American Medical Association projects a shortage of 37 800 to 124 000 physicians by 2034.^1^ Central to this dynamic landscape is the growth of the role of physician assistants and associates^2^ (PAs), who are vital in delivering high-quality patient care. PAs are highly skilled medical professionals who provide a wide range of health care services, working collaboratively with physicians, nurses, and other health care professionals.^3,4^ Since its inception in the 1960s, the PA profession has seen significant growth and recognition, consistently ranking among the top health care jobs. By 2030, it is expected to grow by 27%.^5^

PAs are licensed clinicians who practice medicine across various specialties and settings. They are trusted, rigorously educated health care professionals dedicated to expanding access to care and transforming health and wellness through patient-centered, team-based medical practice. PAs are educated at the master’s degree level, with more than 308 PA programs across the country. These programs typically last approximately 27 months and include classroom instruction as well as over 2000 hours of experiential clinical training (clinical rotations).

Admission to PA programs is highly competitive, requiring a bachelor’s degree and completion of courses in basic and behavioral sciences, with an average prerequisite grade point average (GPA) of 3.0. Prospective students must also have significant direct patient contact experience, averaging over 3000 hours, often gained through roles such as paramedics, athletic trainers, or medical assistants. The application process is rigorous, with 43% of programs requiring the Graduate Record Examination (GRE) and a few programs requiring the Physician Assistant College Admissions Test, a specialized entrance examination for PA applicants.

The cost of applying to PA programs is substantial. The application fee for the national application platform, the Central Application Service for Physician Assistants (CASPA), is $184 for the first program an individual applies to and $61 for each additional program. Once applicants are matriculated, tuition costs remain high: $57 955 for in-state tuition at public universities, $96 171 for out-of-state tuition at public universities, and $100 212 for private universities.^6^ These significant financial barriers can deter many potential applicants, particularly those from underrepresented minority backgrounds, who may already face economic disadvantages.

Despite compelling evidence demonstrating the benefits of a diverse health care workforce, the underrepresentation of minority individuals remains a persistent challenge across various health care professions.^7,8,9,10^ This is particularly concerning given the significant disparities in morbidity and mortality faced by racial and ethnic minority groups.^11^ For example, in 2021, the mean life expectancy at birth for non-Hispanic Black individuals was 70.8 years, notably lower than the 76.4 years reported for non-Hispanic White individuals.^12^ Of particular concern is the disproportionately low life expectancy among Black men, with a mean of about 66.7 years.^12^ Research suggests that when health care practitioners reflect the demographic characteristics, life experiences, values, and culture of their patients, it fosters trust, enhances communication, increases use of health care services, boosts patient satisfaction, and improves patient outcomes.^13,14,15,16,17,18^ Furthermore, evidence suggests that a diverse educational setting exposes students to a variety of perspectives, broadening their understanding and enhancing cognitive skills such as critical thinking and problem-solving.^19,20,21,22,23,24^ This additive enhancement of performance among individuals with high ability has been termed *superadditivity*.^20^ Therefore, addressing the barriers to PA program admission is essential for ensuring an enhanced learning environment and a health care workforce ready to improve the health outcomes for all US residents.

A 2021 study^9^ examined the diversity of workers and recent graduates of the 10 most prominent health professions. Among these, the PA profession ranked last for representation of Black health care workers and in the bottom third for representation among 2019 graduates. The PA profession was established in 1967 and has predominantly consisted of White women. As of 2022, 59.9% of certified PAs were White women, and only 1.1% were Black men.^25^ A similar underrepresentation is seen in the physician workforce, with 2.6%^26^ being Black men, making this a national crisis.^27^ Given that PAs engage in over 500 million patient interactions annually, providing comprehensive care and enhancing access to quality health care,^3^ it is essential to examine the diversity and representation of Black men within the profession. The current state of underrepresentation of Black men poses a significant challenge that hinders the realization of the full potential of our health care system. However, despite acknowledgment of the need for diversity in the profession,^10,28,29,30,31,32,33^ significant improvements are yet to be realized, and some interventions have failed to increase matriculation.^34^

As PA training programs have shown tremendous growth in the past few decades, it is imperative to scrutinize the demographic makeup of PA applicants and matriculants, as well as the systems and barriers that may hinder their increased diversity. Ensuring alignment with the broader goal of cultivating diversity and inclusivity within the health care landscape and reflecting the US demographic composition is crucial. The objective of this study was to examine the pipelines that funnel Black men to the PA workforce. We conceptualize these pipelines as gates, specifically the application gate and the matriculation gate. Using data from prospective PA applicants between 2013 and 2021, this study investigates the historical patterns and US Census parallel representation of Black male applicants and matriculants to PA training programs.

## Methods

### Application and Matriculation Data

This study was considered exempt from approval and the requirement for informed consent by the Charles R. Drew University of Medicine and Science institutional review board because the data were deidentified, in accordance with 45 CFR §46 (2018 Common Rule). We followed the Strengthening the Reporting of Observational Studies in Epidemiology (STROBE) reporting guidelines. The PA Education Association administers CASPA,^35^ a web-based application system that collects comprehensive PA program application information. On March 10, 2023, we obtained individual-level CASPA data from the 2012-2013 to the 2020-2021 application cycles from the PA Education Association. Each CASPA application cycle is named according to the year it begins and ends, opening in April and closing in April of the following year. For simplicity, this report refers to each application cycle by the year it closes. Individuals who receive acceptance to a PA program are classified as matriculants.

In the CASPA application platform, applicants self-reported their gender, racial, and ethnic identity, with the ability to select as many applicable classifications as applicable. For this study, applicants who identified as Black or African American were categorized as Black, regardless of any additional selections. In this analysis, we compared data of applicants and matriculants who selected both Black race and male gender with metrics for all applicants.

### Census Data

The US Census Bureau provides estimates of the national US population overall and stratified by age group and gender, racial, and ethnic identity.^36^ Consistent with the strategy used for the CASPA data, we used estimates determined for all individuals identifying as Black, regardless of any additional racial or ethnic identities. The US Census Bureau denotes these individuals as Black alone or in combination. Not all age groups in the US population are likely to be applying to PA programs. Therefore, we limited the population estimates to the age ranges that constituted the likely potential applicant pool. We determined this age range from the CASPA data as between the 25th and 75th percentile of applicant ages, which corresponded to ages 20 to 29 years. All estimates from the US Census data were then limited to individuals within this age range.

### PA Program Data

The Accreditation Review Commission on Education for the Physician Assistant, Inc, maintains a list of all accredited programs.^37^ Under the assumption that each PA program has been open since it was first accredited, the total number of operating, accredited programs for each year from 2013 to 2021 was calculated.

### Statistical Analysis

Data were analyzed from June 2023 to May 2024. We calculated the frequencies and percentages of applicants and matriculants for the total sample and for Black men for each admission cycle, for cycles ending in years 2013 through 2021. The standardized rates for the total sample and Black men were then calculated as the number of PA program applicants or matriculants divided by the number of estimated individuals in the US aged 20 to 29 years per 100 000 population.

To determine the deficit in the number of applicants and matriculants who were Black men, we first calculated the numbers of those who would be expected to apply and matriculate in the most recent application cycle. Assuming that the probability of an individual applying or matriculating to a PA program is equivalent across groups, the proportional representation of Black men among applicants and matriculants should be equivalent to the proportional representation of Black men aged 20 to 29 years in the US population. We thus calculated the percentage of the US population aged 20 to 29 years in 2021 represented by Black men (8.7%) and multiplied this percentage by the total number of applicants and matriculants to derive the number of applicants and matriculants expected to be Black men. The deficit was then calculated as the difference between the expected number and the actual number. All statistical analyses were conducted using SPSS, version 27 (IBM Corp) and R, version 4.4.1 (R Project for Statistical Computing).

## Results

### Gate 1: Applicants

**Figure 1.**
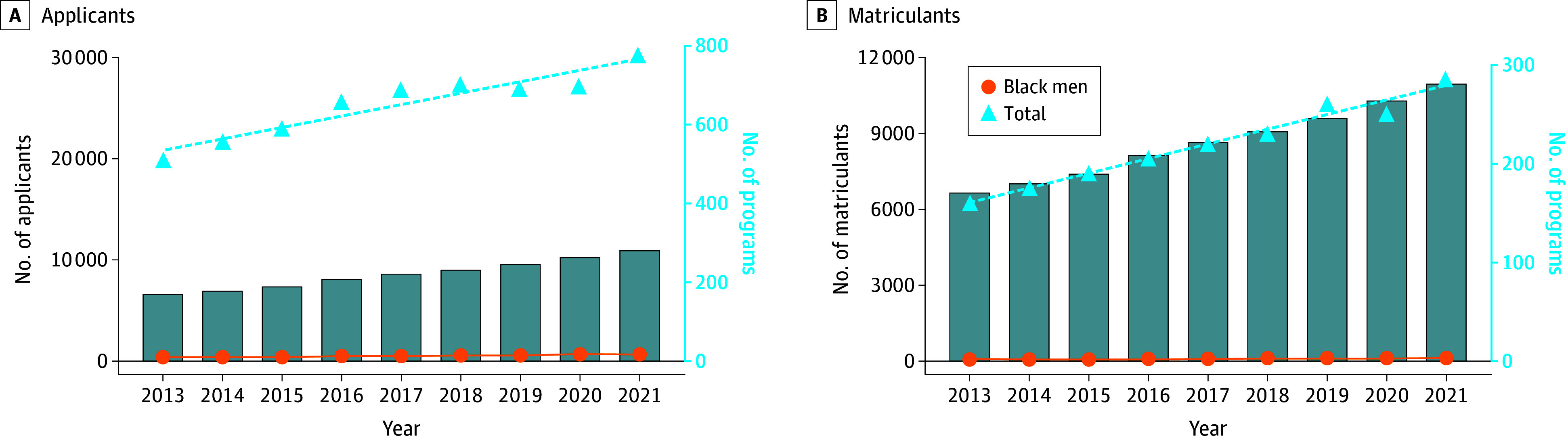
Patterns of Physician Assistant and Associate (PA) Training Programs, Applicants, and Matriculants (2013-2021) Lines indicate a linear best fit. Blue bars represent numbers of active PA programs during the respective year.

**Figure 2.**
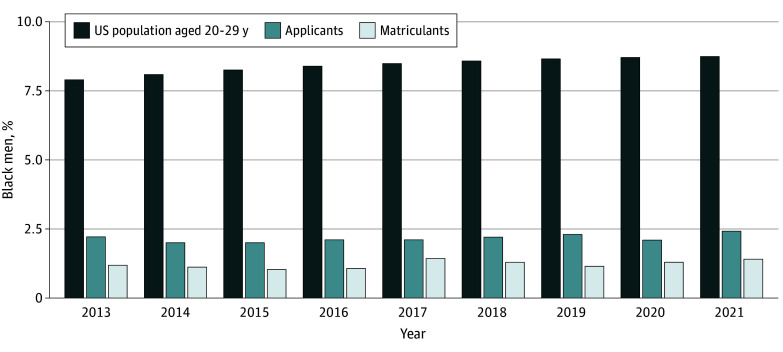
Representation of Black Men in the US Census and Among Physician Assistant and Associate Applicants and Matriculants, 2013 to 2021

Between 2013 and 2021, the total number of applicants increased substantially from 19 761 individuals in 2013 to 30 196 by 2021 (Figure 1 and eTable 1 in Supplement 1). During this same time frame, the cumulative number of accredited PA training programs increased by 64.3% from 171 to 281 (Figure 1). In 2013, applicants who identified as Black men accounted for only 435 of 19 761 applicants (2.2%), a percentage that remained nearly identical for the subsequent 8 application cycles, reaching 732 of 30 196 (2.4%) in 2021 (Figure 2 and eTable 1 in Supplement 1).

**Figure 3.**
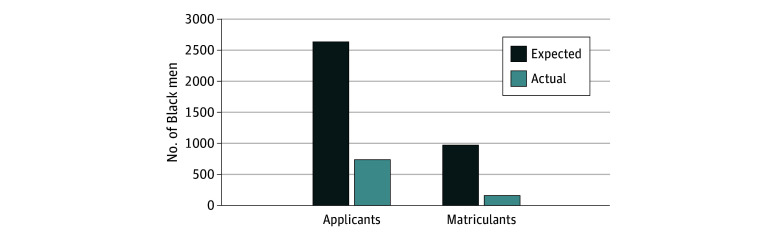
Comparison Between the Number of Actual and Expected Applicants and Matriculants Who Are Black Men in 2021 In 2021, Black men constituted 8.7% of the US population aged 20 to 29 years. Thus, given parity in admissions and matriculation, it is expected that 8.7% of all applicants and matriculants to physician assistant and associate programs should be Black men.

In 2021, overall, 67 applications were received for every 100 000 potential applicants (eTable 2 in Supplement 1). For every 100 000 potential applicants who were Black men, however, only 19 applications were received (eTable 2 in Supplement 1). In 2021, of the total US population aged 20 to 29 years (n = 44 884 060), Black men constituted 8.7% (n = 3 920 231) (Figure 2), so in an equitable scenario, Black men should have represented 8.7% of the 19 761 PA applicants in 2021, for an expected number of 2641 applicants in 308 programs (approximately 9 applicants per program, as opposed to 2) (Figure 3). Given that only 732 applicants in 2021 were Black men, this represents a deficit of 1909 Black male applicants in this cycle alone (Figure 3).

### Gate 2: Matriculants

Between 2013 and 2021, the total number of matriculants also increased from 6192 to 11 115 (Figure 1). However, Black men represented 73 matriculants (1.2%) in 2013 and 156 (1.4%) in 2021 (Figure 2 and eTable 1 in Supplement 1). In 2021, for every 100 000 potential Black men in the US population, only 4 matriculated, whereas 25 matriculated for every 100 000 in the total applicant pool (eTable 2 in Supplement 1). If representation among PA program matriculants was equal to the 8.7% representation among the prospective national pool, we would have expected 972 Black men to have successfully matriculated into 308 PA programs (approximately 3 applicants per program, as opposed to 1 for every 2 programs) (Figure 3). The 156 Black men who matriculated indicate a deficit of 816 Black men matriculating in 2021 (Figure 3).

Although due in part to the lower number of applicants who are Black men, the low representation among matriculants is also affected by the lower matriculation success of these applicants compared with their counterparts. The matriculation rate of Black men increased from 73 of 435 (16.8%) in 2013 to 156 of 732 (21.3%) in 2021 but continued to lag behind the overall matriculation rate for all applicants (11 115 of 30 196 [36.8%]) in 2021 (eTable 1 in Supplement 1). Even given that only 732 Black men applied to PA programs in 2021, if their matriculation rates had been equal to those of the total sample, 269 would have matriculated, for a 172.7% increase of matriculants.

## Discussion

The growth of accredited PA programs by 64.3% from 2013 to 2021, with increases in applicants and matriculants, reflects a growing interest in the PA field. However, several studies^28,29,30,31,32^ have highlighted the underrepresentation of minority applicants and matriculants of all genders in PA training programs. This study is the first, to our knowledge, to assess the representation of Black men specifically, among applicants and matriculants, unmasking the striking underrepresentation. The barriers include a scarcity of Black male applicants and a disproportionately lower matriculation rate. Addressing these issues requires exploring underlying factors and developing strategies to “open the gates” and welcome more Black men into the PA profession.

The underrepresentation of Black men is indicative of broader systemic issues affecting careers in science, technology, engineering, mathematics, and medicine. Barriers include systemic racism, economic disadvantage, and unequal educational opportunities.^38,39,40^ Additional deterrents include high tuition costs, outdated admissions processes, and a lack of role models.

### Opening Gate 1: Increasing the Number of Black Male Applicants

Our results indicate a significant scarcity of Black male applicants to PA programs. To achieve equity, each PA program would need to consider, on average, 9 Black male applicants annually, a substantial increase from about 2 in 2021. Meeting this goal requires targeted campaigns and proactive measures to prepare Black men for PA program applications. These efforts must be ongoing and adaptable to counter barriers and effect lasting change.

Historical data show that personal encounters with PAs impact career choice, with over 40% of PA students motivated by a PA’s care for themselves or family members.^41^ Amplifying the profession’s visibility in Black communities is crucial for opening the application gate for Black male applicants.

Successful applications also require strategy and mentorship. A qualitative study of underrepresented minority students highlighted the value of preapplication mentorship,^42^ while another study identified easily remediable deterrents such as incomplete applications and late submissions.^43^ The competitive nature of PA programs necessitates applying to multiple programs,^44^ but Black prospective students submit fewer applications (median, 4.0; maximum, 56) compared with Asian (median, 7.0; maximum, 118) and White (median, 6.0; maximum, 78) applicants.^45^ PA programs often have additional, unique requirements (eg, the GRE, supplemental application, and in-person interviews) that can disadvantage applicants without mentorship or financial resources for application fees. Additionally, the lack of Black men in the PA workforce leads to a scarcity of mentors, limiting networking opportunities and career guidance.^46^ Mentorship programs can help address these barriers by providing support and advice on navigating the application process, meeting prerequisites, and securing financial aid. Establishing mentorship initiatives connecting applicants with practicing Black male PAs can enhance preparedness and success, fostering a more diverse PA workforce.^39,46^

Compared with medical schools, PA programs lag in using strategies known to increase applicants from minoritized groups, such as minority-focused recruitment and preadmission mentorship.^47^ Enhancing preparedness and support for potential applicants is crucial. Increasing Black male applicants requires a collective effort involving educational leaders, faculty, medical professionals, and community leaders. Partnerships with historically Black colleges and universities and Black organizations, as well as specialized recruitment campaigns, are essential. These initiatives should include informational events, workshops, career fairs, mentor matching, and guidance on educational pathways, CASPA application support, financial aid, and networking opportunities.

### Opening Gate 2: Increasing the Number of Black Male Matriculants

While increasing Black male applicants is essential, it does not guarantee matriculation.^34^ Our results show that in 2021, the matriculation rate for Black men was 21.3%, compared with 36.8% for all applicants. To achieve equity, each PA program needs to matriculate 3 Black men annually, a significant increase from the current rate of 1 per 2 programs.

A 2021 study^44^ reported a positive association between the number of applications submitted and matriculation probability. White and Asian applicants benefit from submitting up to 16 applications. This trend was not reciprocated for Black applicants, among whom there was no added benefit beyond 7 applications.^44^ Consequently, Black applicants face a paradoxical hurdle: they submit fewer applications, reducing their competitive advantage, but increasing the number of applications does not yield the benefit that other demographic groups enjoy. This disparity highlights the complex interplay of systemic barriers and application strategies that uniquely disadvantage Black applicants. Additional huddles at the matriculation gate include heavy reliance on quantitative metrics and insufficient training of admissions decision-makers.^48^ Quantitative metrics like the GRE score and undergraduate GPA can perpetuate biases and inequities.^47,49,50^ Furthermore, quantitative measures may not accurately predict an individual’s ability to thrive in PA training, pass the Physician Assistant National Examination Certifying Examination, or excel in clinical practice.^51^ In contrast, holistic admissions practices that evaluate the whole applicant promote a diverse student body.^47,52,53,54^ While undergraduate GPA has been associated with Physician Assistant National Examination Certifying Examination performance,^55,56,57,58^ additional academic resources during training can counter previous academic deficiencies and ensure success. Others have recommended enrollment in postbaccalaureate academic programs to increase GPA competitiveness, but the financial implications may be a deterrent.^47,59^ In contrast, holistic admissions practices that consider personal attributes such as critical thinking, problem-solving, emotional intelligence, resilience, intellectual curiosity, and communication skills alongside numerical metrics are better predictors of excellence in PA practice but are limited by practical constraints.^21,47,54^ Admissions staff may prioritize quantifiable metrics due to time and resource constraints, potentially disadvantaging Black men who may excel in less quantifiable areas. Furthermore, admissions decision-makers are predominantly not Black, with only 3.1% of PA faculty identifying as Black.^41^ Training admissions faculty to reduce implicit bias is essential.

In addition to revising admissions practices, fostering an inclusive and supportive environment post matriculation is critical. A study revealed that medical students from racial and ethnic minority groups have almost 4 times higher attrition rate compared with students who are not.^60^ Creating a welcoming and supportive environment is vital for attracting and retaining diverse trainees. PA programs should promote diversity through interactive discussions, role-playing, and empathy-building activities to address the unique needs of Black male students.^22,61,62^

### Multiplicative Benefits of Diverse Classrooms

Racial diversity within PA classrooms enhances health care equity. A diverse learning environment enriches education, patient care, and health outcomes. Students bring unique experiences that inspire critical analysis and team-based problem-solving, challenging conventional thinking and improving the educational process.^19,20,21,22,23,24^ Training in a diverse setting prepares practitioners to better serve diverse populations, improving patient outcomes and reducing health disparities.^13,14,16,17,18^ Therefore, increasing the number of Black men in PA programs will yield multiplicative benefits in improving the health of all US residents.

### Navigating Post–Affirmative Action Challenges

The 2023 Supreme Court decision^63^ against affirmative action introduces uncertainty^64^ for admissions practices. Many programs have removed race and ethnicity considerations, raising concerns about the impact on students from racial and ethnic minority groups. Maintaining diversity goals will be challenging. The College Board Education Counsel’s guide^65^ suggests using nonracial parameters like geography and first-generation college status to achieve diversity. The legality of race and ethnicity–based pipeline programs remains unclear. Continued research and adaptive strategies are needed to ensure equitable representation of Black men in PA programs.

### Strengths and Limitations

This study’s novelty lies in assessing Black men’s application and matriculation patterns over 9 years using a national dataset. Using US Census data to calculate rates per 100 000 in the potential applicant pool strengthens our findings.

However, our study has some limitations. First, 2.5% of PA programs with in-house admissions processes were not included. Second, due to the deidentified nature of the CASPA data, all applicants were included in the count for each application cycle without accounting for repeat applicants. Third, our definition of Black race and male gender did not capture the full spectrum of racial and gender identities. Finally, only complete applications in CASPA were considered, omitting incomplete applications, which may signify additional barriers for Black men. These aspects warrant further research.

## Conclusion

This cohort study underscores the urgent need to address the substantial underrepresentation of Black men in the PA profession. This crisis demands a call to action, open discussions, and targeted strategic efforts to ensure equal opportunities for Black men. To achieve population equity, each PA training program should aim to evaluate 9 applications and matriculate 3 Black men. This goal is attainable and represents a crucial step toward enhancing the diversity of the PA workforce and improving the health and well-being of all Americans.

## Notes

Supplement 1. **eTable 1.** Total and Black Male Physician Assistant/Associate Applicants and Matriculants, 2013 to 2021 **eTable 2.** Rate of Total and Black Male Physician Assistant/Associate Applicants and Matriculants, 2013 to 2021

Supplement 2.
Data Sharing Statement
