# Supplementary material for: Underrepresentation of Black Men in Physician Assistant and Associate Training
Source: JAMA Netw Open. 2024 Oct 28;7(10):e2441531. doi: 10.1001/jamanetworkopen.2024.41531 (PMC11519756; doi:10.1001/jamanetworkopen.2024.41531)
Supplement: Supplement 1. — eTable 1. Total and Black Male Physician Assistant/Associate Applicants and Matriculants, 2013 to 2021 eTable 2. Rate of Total and Black Male Physician Assistant/Associate Applicants and Matriculants, 2013 to 2021 [file jamanetwopen-e2441531-s001.pdf]

Supplementary Online Content

Kibe LW, Schrode KM, Paik S, Frias-Sarmiento D. Underrepresentation of Black men in physician assistant/associate training. *JAMA Netw Open*. 2024;7(10):e2441531. doi:10.1001/jamanetworkopen.2024.41531

eTable 1. Total and Black Male Physician Assistant/Associate Applicants and Matriculants, 2013 to 2021

eTable 2. Rate of Total and Black Male Physician Assistant/Associate Applicants and Matriculants, 2013 to 2021

This supplementary material has been provided by the authors to give readers additional information about their work.

© 2024 Kibe LW et al. *JAMA Network Open*.

eTable 1. Total and Black Male Physician Assistant/Associate Applicants and Matriculants, 2013 to 2021

	2013	2014	2015	2016	2017	2018	2019	2020	2021
**Total Sample**									
Applicants (n)	19,761	21,645	22,969	25,594	26,760	27,282	26,855	27,104	30,196
Matriculants (n)	6,192	6,900	7,484	8,062	8,687	8,985	10,149	9,735	11,115
% of applicants that matriculate	31.3	31.9	32.6	31.5	32.5	32.9	37.8	35.9	36.8
**Black Men**									
Applicants (n)	435	430	468	546	572	588	614	578	732
% of all applicants	2.2	2.0	2.0	2.1	2.1	2.2	2.3	2.1	2.4
Matriculants (n)	73	78	77	86	123	116	115	126	156
% of all matriculants	1.2	1.1	1.0	1.1	1.4	1.3	1.1	1.3	1.4
% of applicants who matriculate	16.8	18.1	16.5	15.8	21.5	19.7	18.7	21.8	21.3

eTable 2. Rate of Total and Black Male Physician Assistant/Associate Applicants and Matriculants, 2013 to 2021

	2013	2014	2015	2016	2017	2018	2019	2020	2021
**Total Sample**									
Applicants (rate/100,000)	44.9	48.8	51.2	56.8	59.1	60.1	59.3	60.0	67.3
Matriculants (rate/100,000)	14.1	15.5	16.7	17.9	19.2	19.8	22.4	21.6	24.8
**Black Men**									
Applicants (rate/100,000)	12.5	12.0	12.6	14.4	14.9	15.1	15.6	14.7	18.7
Matriculants (rate/100,000)	2.1	2.2	2.1	2.3	3.2	3.0	2.9	3.2	4.0

Rates were calculated based on national US population estimates of individuals aged 20-29

© 2024 Kibe LW et al. *JAMA Network Open*.
