# Supplementary material for: Underrepresentation of Black Men in Physician Assistant and Associate Training
Source: JAMA Netw Open. 2024 Oct 28;7(10):e2441531. doi: 10.1001/jamanetworkopen.2024.41531 (PMC11519756; doi:10.1001/jamanetworkopen.2024.41531)
Supplement: Supplement 2. — Data Sharing Statement [file jamanetwopen-e2441531-s002.pdf]

Data Sharing Statement

Kibe. Underrepresentation of Black Men in Physician Assistant/Associate Training. *JAMA Netw Open*. Published October 28, 2024. doi:10.1001/jamanetworkopen.2024.41531

Data
Data available: No

Additional Information
Explanation for why data not available: The data belongs to the Physician Assistant Education Association (PAEA). The data can be requested from PAEA. https://paeaonline.org/our-work/research-data-and-support/paea-data-request-and-sharing-policies
